# Early Emergence of HDL Oxidation Across the Body Mass Index Spectrum in Patients with Suspected Coronary Artery Disease

**DOI:** 10.3390/ijms27177570

**Published:** 2026-08-24

**Authors:** Benjamin Sasko, Tonghuan Shi, Martin Christ, Oliver Ritter, Sawa Kostin, Innas Sultana, Simin Delalat, Jörg Celesnik, Metin Senkal, Ibrahim El-Battrawy, Theodoros Kelesidis, Christian Ukena, Nazha Hamdani, Nikolaos Pagonas

**Affiliations:** 1 Ruhr-University Bochum, Marien Hospital Herne, Medical Department II, Hölkeskampring 40, 44625 Herne, Germany; 2 Department of Cardiology, Medical School Theodor Fontane, University Medical Center Brandenburg an der Havel, 14770 Brandenburg an der Havel, Germany; 3 Department of Cellular and Translational Physiology, Institute of Physiology, Molecular and Experimental Cardiology, Institut für Forschung und Lehre (IFL), Ruhr University Bochum, 44801 Bochum, Germany; 4 Department of Cardiology, Knappschaft Kliniken Bottrop, Academic Teaching Hospital, University Duisburg-Essen, 46242 Bottrop, Germany; 5 Faculty of Health Sciences, Joint Faculty of the Brandenburg University of Technology Cottbus—Senftenberg, the (MHB) Theodor Fontane and the University of Potsdam, 14469 Potsdam, Germany; 6 Department of General and Visceral Surgery, Knappschaft Klinik Bottrop, Academic Teaching Hospital, University Duisburg-Essen, 46242 Bottrop, Germany; 7 Department of General and Visceral Surgery, Marien Hospital Witten, 58452 Witten, Germany; 8 Department of Cardiology and Rhythmology St. Josef Hospital, UK RUB, Ruhr University Bochum, 44795 Bochum, Germany; 9 Department of Medicine, Division of Infectious Diseases and Geographic Medicine, University of Texas Southwestern Medical Center, Dallas, TX 75390, USA; 10 Department of Pharmacology and Pharmacotherapy, Center for Pharmacology and Drug Research & Development, HCEMM-SU Cardiovascular Comorbidities Research Group Intézet Címe Semmelweis University Budapest Hungary, 1089 Budapest, Hungary; 11 Department of Physiology Cardiovascular Research Institute Maastricht, Maastricht University, 6229 ER Maastricht, The Netherlands; 12 Department of Cardiology, Medical School Theodor Fontane, University Hospital Ruppin-Brandenburg, 16816 Neuruppin, Germany

**Keywords:** oxidized HDL, cardiometabolic function, oxidative stress, acute coronary syndrome, HDL function

## Abstract

Obesity, commonly defined by body mass index (BMI), promotes cardiovascular disease through chronic inflammation and oxidative stress, which impair high-density lipoprotein (HDL) function and favor oxidative modification. Oxidized HDL (HDLox) may link adiposity to cardiovascular risk. However, the association between BMI and HDLox across the adiposity spectrum remains unclear. We therefore examined this relationship in a Coronary-Suspected Cohort. This study included 1227 consecutive patients undergoing elective coronary angiography. Participants were stratified into five predefined BMI categories. HDLox was quantified using a validated fluorometric assay assessing HDL lipid peroxide content. Associations between BMI and HDLox were analyzed using univariable and multivariable linear regression across the full BMI range and within a restricted BMI interval (15–35 kg/m^2^). HDLox levels were significantly higher in overweight and obese individuals compared with normal-weight participants (*p* = 0.002 and *p* < 0.001). HDLox increased from normal weight to obesity class I (BMI 30–34.9 kg/m^2^) and plateaued at higher BMI levels, indicating a non-linear relationship. In the overall cohort, BMI showed a weak but significant association with HDLox (β = 0.064, *p* = 0.024). Within the restricted BMI interval, the association was more pronounced (β = 0.158, *p* < 0.001). In multivariable models, BMI, high-sensitivity C-reactive protein, and male sex remained independently associated with HDLox, whereas traditional cardiovascular risk factors were not. HDL oxidation increases early during weight gain and may represent a functional marker linking adiposity-related inflammation and oxidative stress to cardiovascular vulnerability.

## 1. Introduction

Obesity has emerged as one of the most prevalent and consequential risk factors for cardiovascular disease worldwide [1,2]. Its contribution to atherosclerosis, coronary artery disease, and heart failure extends beyond the accumulation of excess body weight and encompasses profound metabolic, inflammatory, and vascular alterations [3,4,5,6]. In contemporary cardiology cohorts, obesity frequently coexists with multiple cardiometabolic comorbidities, creating a state of sustained physiological stress that accelerates cardiovascular disease development and progression.

A defining feature of obesity is chronic, low-grade inflammation [4]. Adipose tissue is recognized as an active endocrine organ that secretes pro-inflammatory cytokines, adipokines, and chemokines, thereby promoting systemic inflammation [3,7]. This inflammatory milieu is closely linked to increased oxidative stress, driven by enhanced production of reactive oxygen species and impaired antioxidant defenses. Together, chronic inflammation and oxidative stress represent central mechanisms through which obesity contributes to endothelial dysfunction, impaired vascular repair, and adverse cardiovascular remodeling [4,8]. However, how these systemic disturbances relate to measurable cardiovascular markers remains under investigation.

Lipid metabolism is profoundly altered in obesity, traditionally characterized by hypertriglyceridaemia, reduced high-density lipoprotein cholesterol (HDL-C), and qualitative changes in lipoprotein composition [9,10,11]. Increasing evidence suggests that the cardiovascular relevance of lipoproteins extends beyond their circulating concentrations and depends critically on their functional properties. In this context, HDL plays a particularly important role [12,13]. In addition to its established function in reverse cholesterol transport, HDL exerts anti-inflammatory, anti-oxidative, and endothelial-protective effects, thereby contributing to vascular homeostasis and repair [14,15,16,17,18]. For these reasons, HDL has been conceptualized as a “repair molecule” that counterbalances inflammation- and oxidation-induced vascular injury. Importantly, HDL is highly susceptible to modification under conditions of metabolic stress. Chronic inflammation and oxidative stress can alter HDL structure and composition, leading to impaired anti-oxidative capacity and loss of anti-inflammatory function [19,20]. Oxidative modification of HDL transforms the particle into a dysfunctional form, commonly referred to as oxidized HDL (HDLox) [19,21,22]. HDLox exhibits reduced protective properties and may even acquire pro-inflammatory characteristics, thereby potentially contributing to vascular dysfunction rather than preventing it [19,20]. As such, oxidized HDL serves as an integrative marker of oxidative stress, inflammation, and altered lipoprotein biology. In this context, we have previously demonstrated that oxidized HDL is associated with a broader state of cardiometabolic dysfunction characterized by vascular inflammation, impaired antioxidant defense, and endothelial dysfunction, as reflected by increased adhesion molecule expression, reduced paraoxonase-1 activity, and disrupted nitric oxide bioavailability [23]. Consistent with these mechanistic associations, elevated levels of HDLox have been linked to coronary artery disease and acute coronary syndromes, heart failure with preserved ejection fraction, and atrial fibrillation [24,25,26].

Obesity establishes a pro-inflammatory and pro-oxidative systemic milieu that promotes oxidative modification of HDL particles. Experimental studies and clinical investigations have provided initial evidence that obesity is associated with impaired HDL function [27,28]. Notably, Davidson and colleagues demonstrated higher levels of oxidized HDL in obese individuals, supporting the notion that excess adiposity is associated with HDL oxidative modification [29]. While this study provided important mechanistic insight, its limited sample size precluded a detailed evaluation of the relationship between body mass index (BMI) and HDL oxidation across the full spectrum of adiposity.

Specifically, it remains unclear whether HDL oxidation increases proportionally across the entire BMI spectrum, whether threshold effects exist, or whether HDL dysfunction already emerges in the pre-obese range. It is unclear whether the association between obesity and HDL oxidation follows a linear, dose-dependent pattern across increasing BMI categories or whether HDL oxidation occurs predominantly beyond certain thresholds of adiposity. The relationship between BMI and oxidized HDL has not been systematically examined in large clinical cohorts with substantial cardiometabolic burden, such as those undergoing evaluation for suspected coronary artery disease.

Therefore, the present study investigated the relationship between body mass index and oxidized HDL levels in a large real-world cardiology population. By stratifying participants into established BMI categories ranging from normal weight to severe obesity, we aimed to characterize the distribution of HDLox across increasing degrees of adiposity.

## 2. Results

### 2.1. Demographic Characteristics of Study Candidates

The study population comprised 1277 individuals stratified into five predefined BMI categories: normal weight (BMI < 25 kg/m^2^), overweight (BMI 25–29.9 kg/m^2^), obesity class I (BMI 30–34.9 kg/m^2^), obesity class II (BMI 35–39.9 kg/m^2^), and obesity class III (BMI ≥ 40 kg/m^2^). The majority of participants were classified as overweight or moderately obese, whereas individuals with severe obesity constituted a smaller proportion of the cohort (Table 1 and Table 2).

The median age was comparable across BMI strata, with a tendency toward a slightly younger age among individuals with severe obesity (Table 1 and Table 2). Across increasing BMI categories, a clear gradient in the prevalence of cardiometabolic comorbidities was observed. Already in the pre-obese range, male sex (OR 1.75, 95% CI 1.28–2.38; *p* = 0.001), hypertension (OR 1.56, 95% CI 1.07–2.25; *p* = 0.021), diabetes mellitus (OR 1.61, 95% CI 1.12–2.34; *p* = 0.010), and coronary artery disease (OR 1.62, 95% CI 1.20–2.18; *p* = 0.002) were significantly more prevalent than among normal-weight individuals. These associations were further accentuated in participants with obesity (BMI ≥ 30 kg/m^2^), with markedly higher odds of hypertension (OR 2.11, 95% CI 1.42–3.13; *p* < 0.001) and diabetes mellitus (OR 2.10, 95% CI 1.45–3.04; *p* < 0.001). In contrast, the prevalence of current smoking, hyperlipidaemia, chronic kidney disease, and stroke did not differ significantly across BMI categories (all *p* > 0.3). Atrial fibrillation showed a numerical increase with higher BMI but did not reach statistical significance (OR 1.40, 95% CI 0.97–2.02; *p* = 0.088). Further details are provided in Table 3.

### 2.2. Laboratory Parameters and Metabolic Profile

Laboratory analyses revealed distinct obesity-related metabolic and inflammatory patterns. Markers of glycaemic metabolism, including fasting glucose and HbA1c, increased progressively across BMI categories, demonstrating a clear relationship (*p* < 0.001 for trend), consistent with the rising prevalence of diabetes mellitus. Lipid profiles differed across BMI categories. Compared with normal-weight participants, HDL cholesterol levels were generally lower and triglyceride concentrations generally higher in participants with greater adiposity, although neither parameter changed strictly monotonically across all BMI strata. Total cholesterol and LDL cholesterol showed no consistent differences across BMI categories (both *p* > 0.10), while lipoprotein(a) concentrations varied without a discernible BMI-related pattern (*p* > 0.20).

Inflammatory burden, reflected by high-sensitivity C-reactive protein, increased across BMI categories, indicating progressively enhanced low-grade inflammation with higher adiposity (*p* < 0.001). Renal function, assessed by estimated glomerular filtration rate, exhibited only modest variation across BMI strata (*p* > 0.05), consistent with the largely stable prevalence of chronic kidney disease observed in the clinical analyses. For details, please see Table 1 and Table 2.

Overall, stratification by BMI revealed a progressive accumulation of cardiometabolic risk factors, inflammatory burden, and adverse lipid characteristics with increasing adiposity.

### 2.3. Antioxidant HDL Function in Relation to BMI

**Figure 1:**
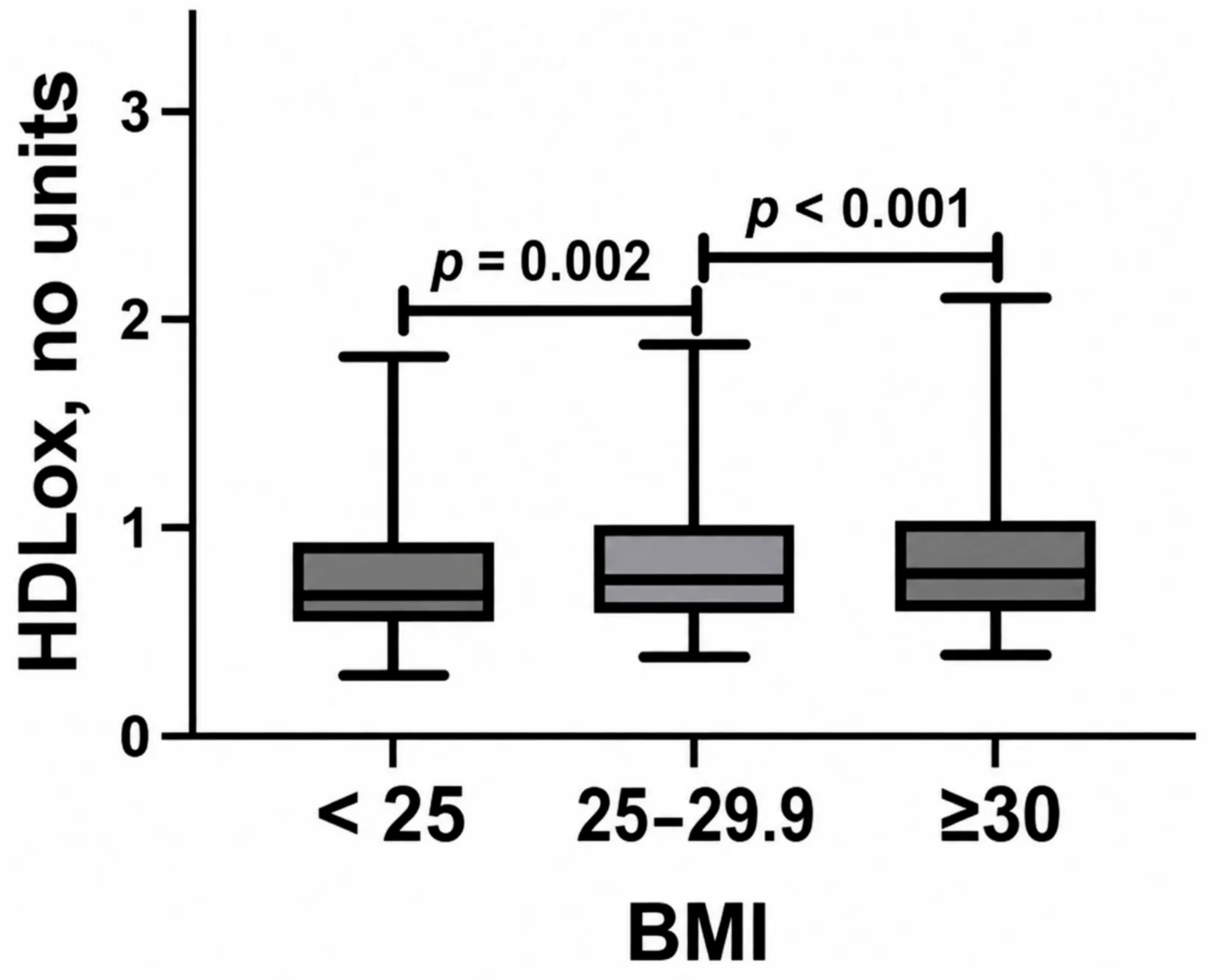
Distribution of oxidized HDL (HDLox) across predefined BMI categories in the study cohort. HDLox levels increased from normal weight to overweight and obesity class I, indicating early HDL oxidation during weight gain. In higher-BMI categories, HDLox levels remained elevated but did not increase further, suggesting a plateau of HDL oxidation at more advanced stages of obesity. Data are presented as median with interquartile range. Statistical comparisons between groups were performed using the Kruskal–Wallis test with post-hoc pairwise comparisons where appropriate.

As shown in Table 1, HDLox levels were significantly higher in individuals with pre-obesity (BMI 25–29.9 kg/m^2^, *p* = 0.002) and obesity (BMI ≥ 30 kg/m^2^, *p* < 0.001) compared with normal-weight participants (BMI < 25 kg/m^2^). HDLox concentrations differed significantly across predefined BMI categories (Table 2 and Figure 1). Median HDLox values increased from the normal-weight group to the overweight group and reached their highest levels in individuals with obesity class I (BMI 30–34.9 kg/m^2^). This increase was already evident within the pre-obese range. In contrast, no further stepwise increase was observed in higher obesity classes; HDLox levels in obesity class II and class III remained elevated but were comparable to those in obesity class I, indicating a plateau at higher degrees of adiposity.

**Figure 2:**
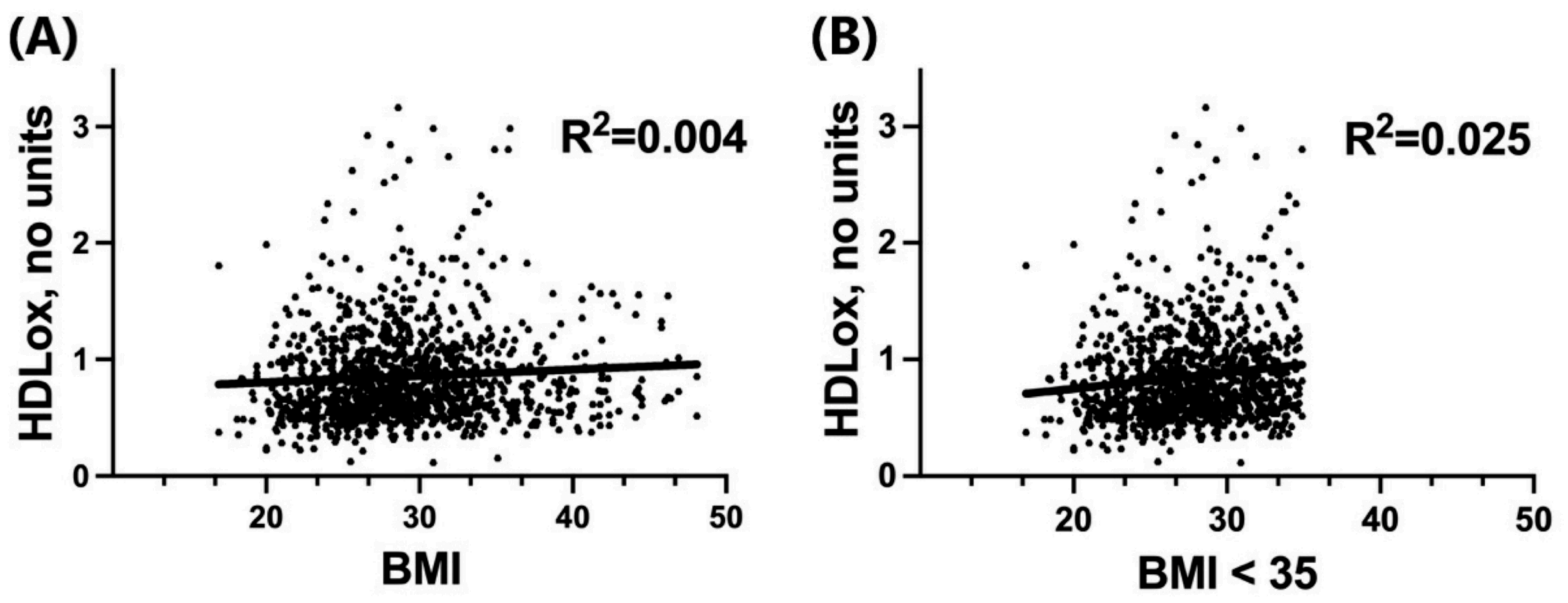
Relationship between body mass index (BMI) and oxidized HDL (HDLox). (**A**) Scatterplot depicting HDL lipid peroxide content (HDLox; no unit) plotted against BMI (kg/m^2^) in the overall cohort (*n* = 1277). The solid line represents the fitted univariable linear regression (HDLox = 0.69 + 5.46 × 10^−3^ × BMI), with the 95% confidence interval indicated by the flanking lines. The linear model explained only a small proportion of variance (R^2^ = 0.004), consistent with a weak overall linear association across the full BMI spectrum. (**B**) Scatterplot illustrating the association between BMI and HDLox within the restricted BMI range of 15–35 kg/m^2^ (*n* = 1080). The solid line represents the fitted univariable linear regression model (HDLox = 0.24 + 0.02 × BMI), with flanking lines indicating the 95% confidence interval. Within this biologically relevant BMI range, the linear association was more pronounced compared with the full cohort analysis (R^2^ = 0.025), reflecting a stronger relationship between increasing adiposity and HDL oxidation before the plateau observed at higher BMI levels.

Across the entire cohort, the association between BMI and HDLox was non-linear, characterized by an increase at lower BMI levels followed by a plateau at higher BMI values (Table 2 and Figure 2). Accordingly, univariable linear regression across the full BMI spectrum showed only a weak, but statistically significant, association between BMI and HDLox (β = 0.064, *p* = 0.024; R^2^ = 0.004). Given the apparent plateau beyond a BMI of approximately 35 kg/m^2^, a secondary restricted-range analysis was performed in participants with BMI between 15 and 35 kg/m^2^. Within this biologically relevant range, BMI was linearly associated with HDLox (β = 0.158, *p* < 0.001), indicating a stronger relationship between adiposity and HDL oxidation at lower to moderate BMI levels.

### 2.4. HDLox in Metabolically and Inflammation-Focused Models

In the overall cohort, a multivariable linear regression model incorporating markers of adiposity, inflammation, and metabolic burden identified several independent determinants of HDLox. Higher BMI remained significantly associated with increased HDLox levels (β = 0.150, *p* = 0.002), alongside systemic inflammation, reflected by hsCRP (β = 0.131, *p* = 0.004), and male sex (β = 0.202, *p* < 0.001). Glycaemic burden, assessed by HbA1c, was also independently associated with HDLox (β = 0.103, *p* = 0.033), although with a smaller effect size. In contrast, age (*p* = 0.222) and the presence of coronary artery disease (*p* = 0.206) were not independently associated with HDLox.

Given the non-linear association observed in univariable analyses, a restricted multivariable model was additionally performed in participants with BMI < 35 kg/m^2^. Within this range, the association between adiposity and HDL oxidation became more pronounced. BMI remained independently associated with HDLox (β = 0.169, *p* = 0.001), together with hsCRP (β = 0.144, *p* = 0.004) and male sex (β = 0.225, *p* < 0.001). In contrast, HbA1c (*p* = 0.156), age (*p* = 0.324), hypertension, and coronary artery disease (all *p* > 0.4) were not independently associated with HDLox. Collectively, these findings indicate that HDL oxidation in the lower-to-moderate adiposity range is primarily driven by adiposity-related inflammatory stress rather than by established cardiovascular disease or advanced metabolic derangement.

### 2.5. Cardiovascular Risk Factor Model

In a multivariable linear regression model incorporating established cardiovascular risk factors in the overall cohort, BMI (β = 0.113, *p* = 0.007) and male sex (β = 0.195, *p* < 0.001) remained independently associated with higher HDLox levels. LDL cholesterol showed an inverse association with HDLox (β = −0.114, *p* = 0.006). In contrast, age, hypertension, smoking status, HbA1c, and the presence of coronary artery disease were not independently associated with HDLox. Overall, this cardiovascular risk factor-based model showed limited explanatory performance, indicating that traditional cardiovascular risk factors capture only a small proportion of the determinants of HDL oxidation.

A similar pattern was observed in a cardiovascular risk factor model restricted to participants with BMI < 35 kg/m^2^. BMI (β = 0.126, *p* = 0.004) and male sex (β = 0.228, *p* < 0.001) remained independently associated with HDLox, while LDL cholesterol again showed an inverse association (β = −0.120, *p* = 0.007). Other cardiovascular risk factors, including age, hypertension, smoking status, HbA1c, and coronary artery disease, were not independently related to HDLox, underscoring the limited ability of classical cardiovascular risk profiles to explain HDL oxidation.

### 2.6. HDLox in Relation to BMI and Coronary Artery Disease (CAD)

**Figure 3:**
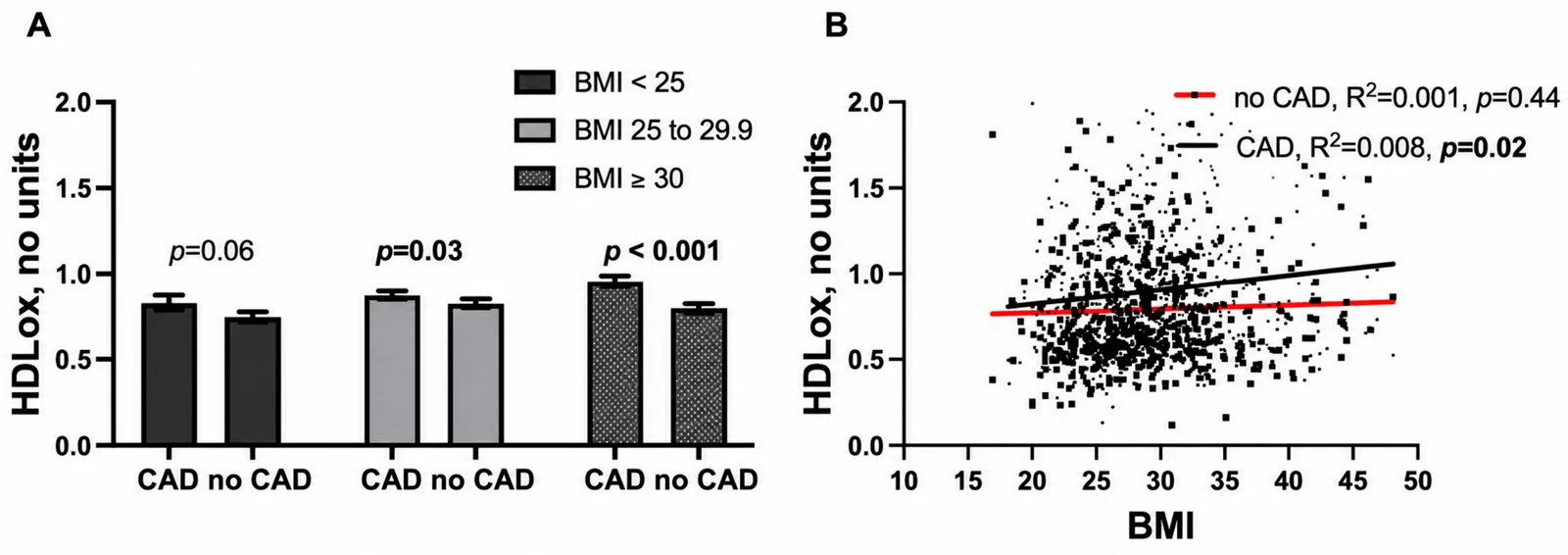
Relationship between body mass index (BMI), oxidized HDL (HDLox), and coronary artery disease (CAD). (**A**) Distribution of HDLox levels across BMI categories stratified by the presence or absence of CAD. Within both the pre-obese (BMI 25–29.9 kg/m^2^) and obese (BMI ≥ 30 kg/m^2^) groups, individuals with CAD exhibited higher HDLox levels compared with those without CAD. Data are presented as median with interquartile range. Group comparisons were performed using non-parametric tests as appropriate. (**B**) Scatterplots illustrating the association between BMI and HDLox stratified by CAD status. Separate univariable linear regression lines are shown for individuals with and without CAD, with shaded areas indicating 95% confidence intervals. A significant positive association between BMI and HDLox was observed within the CAD subgroup, whereas the association was weaker in individuals without CAD. The proportion of explained variance was low in both groups, as reflected by small R^2^ values.

As shown in Figure 3A,B, HDLox levels were higher in individuals with CAD compared with those without CAD within both the pre-obese (BMI 25–29.9 kg/m^2^) and obese (BMI ≥ 30 kg/m^2^) categories. In Figure 3B, linear regression analyses demonstrated a significant positive association between BMI and HDLox within the CAD group, whereas this association was weaker and not consistently significant in individuals without CAD. However, BMI explained only a small proportion of the variance in HDLox, as indicated by the low R^2^ values. These cross-sectional findings demonstrate differences in the BMI–HDLox association according to prevalent CAD status but do not establish a temporal or causal relationship among BMI, HDLox, and CAD.

### 2.7. Determinants of HDL Oxidation in the Pre-Obesity Subgroup (BMI 25–29.9 kg/m^2^)

Within the pre-obese range, HDLox levels were higher than in normal-weight participants but lower than in those with obesity (Table 1 and Table 2). In univariable analysis, BMI was positively associated with HDLox (standardized β = 0.098, *p* = 0.006). After multivariable adjustment, BMI was no longer associated with HDLox. In the metabolic and inflammatory model, hsCRP (β = 0.414, *p* < 0.001), HbA1c (β = 0.154, *p* = 0.027), and male sex (β = 0.178, *p* = 0.009) were independently associated with higher HDLox. In the cardiovascular risk factor model, only male sex remained independently associated with HDLox (β = 0.204, *p* = 0.002), while HbA1c and coronary artery disease showed non-significant trends. Overall, these findings suggest that HDL oxidation in pre-obesity is more closely related to inflammatory and metabolic alterations than to BMI itself or traditional cardiovascular risk factors. Complete regression results are presented in Table 4.

## 3. Discussion

In this large clinical cohort, we demonstrate that dysfunctional, oxidized HDL is closely linked to adiposity and systemic inflammation, while being only partially explained by traditional cardiovascular risk factors. Our findings reveal a non-linear relationship between BMI and HDL oxidation. Notably, HDLox levels are already elevated in individuals classified as overweight (BMI 25–29.9 kg/m^2^), increase further with moderate obesity (BMI 30–34.9 kg/m^2^), and subsequently plateau at higher BMI levels. This pattern indicates a critical window during which HDL dysfunction emerges early in the course of weight gain and does not increase proportionally with more advanced stages of obesity.

Across the full BMI spectrum, univariable analyses showed only a weak linear association between BMI and HDLox. However, restriction to biologically relevant BMI ranges uncovered a more distinct relationship. In individuals with BMI below 35 kg/m^2^, BMI was robustly and independently associated with HDLox, suggesting that increasing adiposity exerts its strongest impact on HDL function at early to moderate stages of weight gain. This plateau may reflect a ceiling effect in oxidative modification capacity, beyond which additional adiposity no longer translates into proportionally higher lipid peroxidation of HDL particles.

HDL is increasingly recognized not merely as a cholesterol carrier but as a dynamic functional particle whose antioxidative, anti-inflammatory, and vasoprotective properties depend on its structural integrity [14,15,16,17,18]. Obesity-associated chronic low-grade inflammation and oxidative stress induce qualitative remodeling of HDL particles, including triglyceride enrichment, altered apolipoprotein composition, and displacement of protective enzymes such as paraoxonase-1 (PON-1) [9,28,29,30], changes that impair antioxidant capacity and increase susceptibility to oxidative modification. Importantly, such functional impairment may occur despite relatively preserved HDL cholesterol levels, underscoring the dissociation between HDL quantity and HDL quality [31].

Expanding adipose tissue actively interacts with circulating lipoproteins and modulates cholesterol flux, as demonstrated in experimental models [32]. Early adipocyte hypertrophy is accompanied by inflammatory activation and increased production of reactive oxygen species [33,34,35,36,37]. Within this pro-oxidative milieu, structurally remodeled HDL particles become more vulnerable to lipid peroxidation, consistent with the elevated HDLox levels observed in our cohort and prior mechanistic work linking HDL oxidation to reduced antioxidant capacity [30,31,32,36,37].

In the presence of adiposity-associated low-grade inflammation and oxidative stress, such structurally and functionally altered HDL particles may be particularly prone to oxidation, consistent with the elevated HDLox levels observed in our cohort [30].

A particularly important finding of the present study is that HDL oxidation is already significantly increased in the pre-obese range, indicating that qualitative HDL dysfunction emerges early during weight gain. This observation is biologically plausible in light of adipose tissue biology at early stages of adiposity expansion [36,37]. Pre-obesity should not be regarded as a metabolically neutral state, as even modest increases in fat mass are accompanied by early adipocyte hypertrophy, altered adipokine secretion, and the initiation of low-grade inflammatory processes within adipose tissue [33,34,35,36,38,39]. Experimental and translational studies have demonstrated that early adipose tissue expansion is associated with macrophage infiltration and increased release of proinflammatory cytokines and reactive oxygen species, well before overt obesity or clinically apparent metabolic disease develops [40]. These early inflammatory and oxidative signals create a systemic environment capable of directly affecting circulating lipoproteins.

Across all metabolically focused regression models conducted in our study, systemic inflammation emerged as a central determinant of HDL oxidation. hsCRP consistently showed independent associations with HDLox in both the overall cohort and BMI-restricted analyses, underscoring the close interplay between low-grade inflammation and HDL dysfunction. In the pre-obese subgroup, markers of early metabolic stress—particularly HbA1c—were strongly associated with HDLox, whereas BMI itself lost independent significance after adjustment. This pattern suggests that, at early stages of adiposity, metabolic and inflammatory pathways mediate the adverse effects of weight gain on HDL quality before overt obesity is established. This interpretation is further supported by experimental and proteomic studies demonstrating that inflammatory and metabolic stress remodel HDL composition and function early during weight gain, impairing cholesterol efflux capacity and antioxidant properties prior to the development of overt obesity or dyslipidaemia [41,42].

Experimental evidence from diet-induced obesity models further indicates that pro-inflammatory, immune-driven remodeling of the HDL proteome occurs early during weight gain, preceding the development of hepatic steatosis, dyslipidaemia, and overt metabolic dysfunction [40]. Collectively, these findings highlight the time-dependent nature of cardiometabolic alterations and position HDL oxidation as an early marker of adiposity-related metabolic and inflammatory stress. Their relationship with clinically manifest cardiovascular disease is therefore of particular interest.

Notably, the association between BMI and HDLox reached statistical significance within the CAD subgroup (Figure 3B), but this finding reflects an unadjusted within-group relationship rather than an independent effect of CAD. In multivariable analyses, CAD itself was not independently associated with HDLox, suggesting that the observed association is driven by shared underlying mechanisms such as inflammation and metabolic dysregulation. Importantly, this does not contradict previous studies [24,26], which demonstrated higher HDLox levels in patients with CAD. Rather, the present findings extend these observations by indicating that elevated HDLox in CAD likely reflects the accumulation of upstream cardiometabolic and inflammatory processes that characterize both obesity and atherosclerotic disease. Thus, HDL oxidation appears to be more closely linked to the underlying pathophysiological milieu than to the presence of clinically manifest CAD itself, supporting its role as an early and integrative marker of cardiometabolic risk.

Beyond the observed alterations in HDL, the present study also identified changes in the broader lipid profile across BMI categories. The metabolic and lipid parameters did not change monotonically, with deviations particularly evident in the BMI 35.0–39.9 kg/m^2^ group. This finding may reflect the metabolic heterogeneity within BMI strata, as BMI does not directly capture visceral adiposity, insulin resistance, or lipoprotein remodeling [43,44]. Detailed lipoprotein profiling has shown that metabolic health and insulin resistance are associated with the size and subclass distribution of VLDL, LDL, and HDL particles, even among individuals within the same BMI category [43,45]. In particular, increased concentrations of triglyceride-rich VLDL and small dense LDL particles may occur without a corresponding increase in LDL cholesterol and may therefore not be fully reflected by conventional lipid measurements [44,45]. Accordingly, the observed fluctuations in HDL cholesterol and triglycerides and the absence of a clear LDL-cholesterol gradient may reflect differences in metabolic phenotype and lipoprotein composition that are not captured by BMI alone.

Furthermore, the inverse association between LDL cholesterol and HDLox observed in the cardiovascular risk factor models requires cautious interpretation. Participants with established CAD or a greater cardiovascular risk burden were more likely to receive lipid-lowering therapy and may consequently have had lower LDL cholesterol levels despite greater underlying disease burden. Thus, this finding may reflect treatment-related or residual confounding rather than a direct inverse biological relationship between LDL cholesterol and HDL oxidation. The cross-sectional design does not permit further assessment of the direction of this association.

Taken together, our findings suggest that oxidized HDL may not merely act as a passive marker of adiposity-related metabolic and inflammatory stress but could also contribute to obesity-related cardiovascular dysfunction. The early increase in HDL oxidation in the pre-obese range, its non-linear behavior across BMI categories, and its close association with systemic inflammation indicate that qualitative HDL dysfunction emerges early during weight gain. Importantly, oxidative modification of HDL not only reflects inflammatory stress but also leads to a loss of its intrinsic antioxidative, anti-inflammatory, and vasoprotective properties. As a consequence, HDL may lose its physiological repair and protective functions and acquire potentially harmful characteristics, thereby amplifying metabolic and vascular injury as already shown in prior research [24,25,26]. The limited explanatory contribution of traditional cardiovascular risk factors further underscores that HDL oxidation captures a distinct biological process that precedes overt obesity and manifest atherosclerotic disease. This dual role of HDLox—as both an early indicator and a potential mediator of adiposity-related cardiometabolic dysfunction—has important implications for risk stratification and therapeutic targeting, emphasizing the need to move beyond static lipid concentrations toward functional assessments of HDL biology.

## 4. Limitations

Several limitations of this study should be acknowledged. First, the observational and cross-sectional design precludes causal inference. While our findings demonstrate robust associations between adiposity, systemic inflammation, and HDL oxidation, we cannot determine whether HDL oxidation is a cause or a consequence of metabolic and inflammatory stress. Second, HDL oxidation was assessed using a single functional assay, which captures an important aspect of HDL quality but does not comprehensively reflect the full spectrum of HDL functionality. Third, although we adjusted for multiple metabolic and cardiovascular covariates, residual confounding cannot be excluded. In particular, detailed measures of body fat distribution, such as visceral adiposity or ectopic fat depots, were not available and may have provided a more precise assessment of adiposity-related inflammatory burden than BMI alone. Although patients with overt inflammatory diseases were excluded, medication use (e.g., statins and anti-inflammatory agents) may influence HDL oxidation and could therefore represent a source of residual confounding. Fourth, the study cohort consisted of patients undergoing coronary angiography, which may limit generalizability to the broader population. However, this well-characterized clinical cohort also represents individuals at increased cardiometabolic risk, in whom early alterations in HDL function are of particular relevance. Finally, an additional limitation relates to the sex distribution of the study population. The cohort consisted predominantly of male participants, reflecting the clinical referral pattern of patients undergoing coronary angiography for suspected coronary artery disease. As sex-specific differences in HDL composition and function have been reported, the generalizability of our findings to female populations may be limited.

## 5. Methods

### 5.1. Study

This cross-sectional observational study included a clinical cohort recruited at the University Hospitals of Brandenburg and Bochum between 2017 and 2019 [24,25]. Adult patients were consecutively enrolled at the time of elective coronary angiography, which was performed solely on the basis of routine clinical indications and independently of study participation. Eligible patients had known or suspected coronary artery disease (CAD), with or without additional cardiovascular risk factors, and a clinical indication for elective coronary angiography. Exclusion criteria were age < 18 years, active cancer, acute infectious disease, and known rheumatic disease. Based on the angiographic findings, CAD was defined as at least one coronary artery stenosis ≥ 50%; patients without coronary stenosis or with stenoses < 50% were classified as having no CAD. Patients were stratified into five predefined body mass index (BMI) categories: normal weight (BMI < 25 kg/m^2^), overweight (BMI 25–29.9 kg/m^2^), obesity class I (BMI 30–34.9 kg/m^2^), obesity class II (BMI 35–39.9 kg/m^2^), and obesity class III (BMI ≥ 40 kg/m^2^).

Patients were excluded if they had a history of malignant disease, evidence of acute infection, or diagnosed rheumatic or inflammatory disorders or were younger than 18 years. Only individuals meeting all eligibility criteria were included in the present analysis.

All participants provided written informed consent. The study was approved by the local ethics committees of the Medical Association of Brandenburg (Nr. AS69bB/2016, 2 June 2016) and the Ruhr University of Bochum (Nr. 15-5279, 23 March 2015) in accordance with the Declaration of Helsinki. The data supporting the findings of this study are available from the corresponding author upon reasonable request. The study has been registered in the German Clinical Trials Register (trial registration number DRKS00014037).

### 5.2. Biomarker and Laboratory Assessment

Fasting blood samples were collected from the elective patients before they underwent any procedures. Isolated serum from blood samples was cryopreserved (−80 °C). Grossly hemolyzed serum was excluded from further analysis.

Standard clinical assays in the central laboratory unit of the university hospitals were performed to obtain laboratory parameters. High-sensitivity C-reactive protein (hs-CRP) was measured using the Roche Tina-Quant CRP (Latex) kit (Roche Diagnostics, Basel, Switzerland).

### 5.3. Assessment of Lipid Peroxide Content of HDL (HDLox)

HDL_ox_ levels in serum were measured using a modified version of a validated fluorometric biochemical cell-free assay. This method detects HDL lipid peroxide content by tracking the oxidation of the fluorochrome Amplex Red [46,47]. The Amplex Red reagent was obtained from the Amplex Red Cholesterol Assay Kit (catalog no. A12216; Life Technologies [Invitrogen], Grand Island, NY, USA). Initially, polyethylene glycol (PEG) precipitation was used to deplete apolipoprotein B (ApoB) from serum. After adding 50 μL of ApoB-depleted serum to each well of a 96-well plate in duplicate, 0.075 units of horseradish peroxidase (HRP) plus 50 µM Amplex Red reagent was applied to each well to reach a total volume of 100 µL. HRP catalyses the reaction between Amplex Red and resorufin when it is coupled with endogenous peroxides. After 1 h of incubation, a Spark 10 M microplate reader (Tecan, Grödig, Austria) was utilized to determine the fluorescence of resorufin at 535/590 nm wavelengths. To normalize and minimize experimental variability across each plate, ApoB-depleted sera from 10 healthy volunteers (who were not participants in the study) were employed as experimental controls. Mean fluorescence from each sample was normalized by the mean fluorescent readout of the pooled control and HDL-C using the following calculation: “normalized” oxidized HDL (nHDLox) = [HDLox_sample × 47 (mg/dL)]/[HDLox_control × HDL-C sample (mg/dL)], where 47 mg/dL represents HDL-C of the pooled serum control. Samples were analyzed after the recruitment of the last patient. The intra-assay coefficient of variation (CV) was 6.7%. The inter-assay CV was 3.7%. All laboratory measurements were performed in Brandenburg, Germany.

### 5.4. Statistical Analysis

Initial analyses evaluated the association between BMI and HDLox across the full BMI spectrum using univariable linear regression, treating BMI as a continuous variable. In addition, HDLox levels were descriptively compared across predefined BMI categories to capture potential threshold effects and clinically relevant strata, including normal weight (BMI < 25 kg/m^2^), pre-obesity (BMI 25–29.9 kg/m^2^), and obesity (BMI ≥ 30 kg/m^2^). This stratified approach allowed assessment of changes in HDL oxidation as early as in the pre-obese range.

Data distribution was assessed using the Shapiro–Wilk test. When normality was not rejected (*p* > 0.10), group comparisons were performed using Student’s *t*-test for two groups or one-way ANOVA for three or more groups. In cases of non-normal distribution, the Mann–Whitney U test was applied for two-group comparisons and the Kruskal–Wallis test for comparisons involving more than two groups. Continuous variables are presented as median with interquartile range, unless stated otherwise. Categorical variables were compared using the χ^2^ test or Fisher’s exact test, as appropriate.

Visual inspection of scatterplots and regression diagnostics indicated a non-linear relationship between BMI and HDLox, characterized by an increase in HDLox at lower BMI values followed by a plateau at higher levels of adiposity. Based on this observation, a secondary restricted-range analysis was performed, limiting the sample to individuals with BMI between 15 and 35 kg/m^2^. This range was selected a priori to reflect a biologically plausible window in which increasing adiposity is most likely to translate into incremental oxidative modification of HDL. Within this restricted BMI range, univariable and multivariable linear regression analyses were conducted to assess the presence and robustness of linear associations between BMI and HDLox. Two prespecified multivariable linear regression models were used to assess factors associated with HDLox. The metabolic and inflammatory model included BMI, hsCRP, HbA1c, sex, age, hypertension, and coronary artery disease. The cardiovascular risk factor model included BMI, HbA1c, sex, age, LDL cholesterol, hypertension, current smoking, and coronary artery disease. The same model structures were used in the overall cohort and in the restricted BMI subgroup analyses. All multivariable analyses were conducted using complete cases for the variables included in the respective model. Standardized regression coefficients (β), 95% confidence intervals, and *p*-values are reported.

The restricted-range analysis was performed as a sensitivity and exploratory approach to further characterize the observed non-linear association and was interpreted in conjunction with the primary analyses across the full BMI spectrum. Results from this analysis were not considered confirmatory but were used to support biological plausibility and guide interpretation of the main findings.

All *p*-values are two-sided, and values <0.05 were considered statistically significant. An experienced statistician performed all statistical analyses using the SAS/STAT version 15.4 (SAS Institute Inc., Cary, NC, USA) and GraphPad Prism version 8 for figures (GraphPad Software, San Diego, CA, USA).

## 6. Conclusions

The assessment of oxidized HDL in the context of adiposity and inflammation provides additional insight into the associations among obesity, HDL function, and cardiovascular health. In this cross-sectional study, HDL oxidation was associated with adiposity and systemic inflammation, with higher levels already observed in the pre-obese range and with a plateau at higher BMI levels. The multivariable findings further indicate that HDLox is more closely related to inflammatory and metabolic characteristics than to traditional cardiovascular risk factors.

These findings support consideration of HDL quality alongside conventional quantitative HDL measures. HDLox may provide complementary biological information beyond HDL-C levels and established cardiovascular risk factors, particularly in individuals at early stages of increased adiposity. However, owing to the cross-sectional design of the study, the present findings do not establish the temporal sequence or causal mechanisms underlying the observed associations.

Pre-obesity may represent a relevant stage for further investigation, as increased HDL oxidation was already detectable before the threshold of manifest obesity. Longitudinal and mechanistic studies are required to determine whether HDL oxidation contributes to metabolic and cardiovascular disease development, serves as a marker of underlying inflammatory and metabolic stress, or represents both processes.

## Figures and Tables

**Table 1:** Baseline characteristics of all participants. Data are presented as median (interquartile range) for continuous variables and number (percentage) for categorical variables. Participants were categorized into three groups: no obesity (BMI < 25 kg/m^2^), pre-obesity (BMI 25–29.9 kg/m^2^), and obesity (BMI ≥ 30 kg/m^2^). *p*-value ^1^ refers to comparisons between no obesity and pre-obesity, and *p*-value ^2^ refers to comparisons between no obesity and obesity.

	No Obesity (BMI < 25)	Pre-Obesity (BMI 25–29.9)	Obesity (BMI ≥ 30)	*p*-Value ^1^	*p*-Value ^2^
*n*	257	520	450		
Age, years	68 (58–76)	68 (59–76)	66 (58–75)	0.52	0.9
Male, no (%)	143 (56)	357 (69)	286 (63)	0.001	0.02
Hypertension, no (%)	195 (76)	432 (83)	391 (86)	0.021	<0.001
Diabetes, no (%)	49 (19)	143 (28)	149 (33)	0.01	<0.001
Current smoking, no (%)	79 (31)	151 (29)	124 (27)	0.6	0.38
HLP, no (%)	187 (73)	378 (73)	337 (74)	0.99	0.65
CKD, no (%)	67 (26)	137 (26)	118 (26)	0.95	0.96
Atrial fibrillation, no (%)	53 (21)	117 (22)	105 (23)	0.55	0.088
Stroke, no (%)	20 (8)	42 (8)	36 (8)	0.9	0.92
CAD, no (%)	132 (51)	328 (63)	268 (59)	0.002	0.1
BMI, kg/m^2^	23 (21–24)	27 (26–29)	33 (31–36)	<0.001	<0.001
nHDL_ox_, no unit	0.67 (0.54–0.93)	0.75 (0.59–1.0)	0.78 (0.6–1.03)	0.002	<0.001
HDL-C, mg/dL	57 (45–68)	49 (41–61)	47 (39–59)	<0.001	<0.001
LDL-C, mg/dL	110 (84–141)	111 (86–143)	111 (86–138)	0.86	0.9
Cholesterol, mg/dL	187 (156–218)	182 (152–216)	181 (153–211)	0.38	0.24
Triglyceride, mg/dL	112 (82–149)	120 (90–175)	132 (99–191)	<0.01	<0.001
Lipoprotein a, mmol/L	19 (7–81)	14 (7–46)	12 (6–68)	0.4	0.1
HbA1c, mmol/mol	5.5 (5.3–6.0)	5.7 (5.4–6.3)	5.9 (5.5–6.8)	<0.001	<0.001
NTproBNP, pg/mL	219 (84–754)	240 (88–696)	176 (79–698)	0.83	0.24
eGFR, mL/min/1.73 m^2^	77 (58–89)	74 (60–89)	74 (58–90)	0.82	0.9
Albumin mg/g Creatinin	4 (2–15)	4 (2–13)	7 (2–24)	0.81	0.02
hsCRP, mg/dL	0.07 (0.00–0.47)	0.1 (0–0.61)	0.13 (0.00–0.91)	0.36	0.02
Glucose, mg/dL	90 (83–103)	101 (88–121)	106 (92–146)	<0.001	<0.001

BMI, body mass index; CAD, coronary artery disease; CKD, chronic kidney disease; HLP, hyperlipidaemia defined as elevated LDL-C, total cholesterol, or triglycerides; HDL, high-density lipoprotein cholesterol; LDL, low-density lipoprotein cholesterol; nHDLox, normalized HDL oxidation; HbA1c, glycated hemoglobin; NTroBNP, N-terminal pro-B-type natriuretic peptide; eGFR, estimated glomerular filtration rate; hsCRP, high-sensitivity C-reactive protein.

**Table 2:** Baseline characteristics of all participants, stratified according to BMI. Data are presented as median (interquartile range) or number (%).

	BMI < 25	BMI 25–29.9	BMI 30–34.9	BMI 35–39.9	BMI ≥ 40
*n*	257	520	303	93	54
Age, years	68 (58–76)	68 (59–76)	66 (58–75)	67 (61–73)	63 (54–71)
Male, no (%)	143 (56)	357 (69)	207 (67)	51 (55)	28 (52)
Hypertension, no (%)	195 (76)	432 (83)	263 (85)	81 (87)	47 (87)
Diabetes, no (%)	49 (19)	143 (28)	94 (31)	38 (41)	17 (32)
Current smoking, no (%)	79 (31)	151 (29)	81 (26)	25 (27)	18 (33)
HLP, no (%)	187 (73)	378 (73)	224 (74)	72 (77)	38 (70)
CKD, no (%)	67 (26)	137 (26)	76 (25)	27 (29)	15 (28)
Atrial fibrillation, no (%)	53 (21)	117 (22)	74 (24)	24 (26)	22 (41)
Statins, no (%)	147 (57)	330 (64)	189 (61)	58 (62)	32 (59)
CAD, no (%)	132 (51)	328 (63)	183 (60)	54 (58)	24 (44)
BMI, kg/m^2^	23 (21–24)	27 (26–29)	32 (30–33)	37 (35–38)	42 (41–45)
nHDL_ox_, no unit	0.67 (0.54–0.93)	0.75 (0.59–1.0)	0.79 (0.6–1.06)	0.73 (0.55–0.95)	0.73 (0.61–1.03)
HDL-C, mg/dL	57 (45–68)	49 (41–61)	47 (39–58)	49 (39–62)	44 (37–59)
LDL-C, mg/dL	110 (84–141)	111 (86–143)	112 (86–140)	104 (85–127)	114 (85–141)
Cholesterol, mg/dL	187 (156–218)	182 (152–216)	184 (154–210)	175 (152–213)	186 (150–215)
Triglyceride, mg/dL	112 (82–149)	120 (90–175)	132 (98–191)	126 (95–174)	155 (111–217)
Lipoprotein a, mmol/L	19 (7–81)	14 (7–46)	14 (6–43)	9 (5–82)	10 (7.5–121)
HbA1c, mmol/mol	5.5 (5.3–6.0)	5.7 (5.4–6.3)	5.8 (5.5–6.5)	6.1 (5.7–7.9)	6.0 (5.6–6.4)
NTproBNP, pg/mL	219 (84–754)	240 (88–696)	169 (80–537)	239 (78–783)	251 (62–1326)
eGFR, mL/min/1.73 m^2^	77 (58–89)	74 (60–89)	74 (57–90)	71 (55–87)	83 (63–94)
Albumin mg/g Creatinin	4 (2–15)	4 (2–13)	7 (2–24)	4 (2–18)	10 (2–35)
hsCRP, mg/dL	0.07 (0.00–0.47)	0.1 (0–0.61)	0.1 (0–0.87)	0.3 (0–0.79)	0.2 (0.03–1)
Glucose, mg/dL	90 (83–103)	101 (88–121)	103 (90–128)	121 (97–205)	114 (99–160)

BMI, body mass index; CAD, coronary artery disease; CKD, chronic kidney disease; HLP, hyperlipidaemia defined as elevated LDL-C, total cholesterol, or triglycerides; HDL, high-density lipoprotein cholesterol; LDL, low-density lipoprotein cholesterol; nHDLox, normalized HDL oxidation; HbA1c, glycated hemoglobin; NTroBNP, N-terminal pro-B-type natriuretic peptide; eGFR, estimated glomerular filtration rate; hsCRP, high-sensitivity C-reactive protein.

**Table 3:** Associations of BMI categories with cardiometabolic comorbidities. Odds ratios (ORs) are presented with 95% confidence intervals (CIs), using participants with a BMI < 25 kg/m^2^ as the reference group.

	BMI 25–29.9 OR (95% CI), *p*-Value	BMI ≥ 30 OR (95% CI), *p*-Value
*n*	520	450
Male sex	1.75 (1.28–2.38); *p* < 0.001	1.39 (1.02–1.90); *p* = 0.039
Hypertension	1.56 (1.08–2.25); *p* = 0.017	2.11 (1.42–3.13); *p* < 0.001
Diabetes	1.61 (1.12–2.32); *p* = 0.011	2.10 (1.45–3.04); *p* < 0.001
CAD	1.62 (1.20–2.19); *p* = 0.002	1.31 (0.96–1.78); *p* = 0.088
Current smoking	0.92 (0.67–1.28); *p* = 0.625	0.92 (0.67–1.28); *p* = 0.625
HLP	1.00 (0.71–1.39); *p* = 0.983	1.08 (0.76–1.52); *p* = 0.672
CKD	1.01 (0.72–1.43); *p* = 0.934	1.01 (0.71–1.43); *p* = 0.965
Atrial fibrillation	1.12 (0.78–1.61); *p* = 0.552	1.40 (0.97–2.02); *p* = 0.088
Stroke	1.72 (0.72–4.11); *p* = 0.221	1.65 (0.68–4.03); *p* = 0.270

**Table 4:** Univariable and multivariable determinants of HDL in pre-obesity. Standardized regression coefficients (β), 95% confidence intervals (CIs), and *p*-values were derived from linear regression analyses restricted to participants with a BMI of 25–29.9 kg/m^2^.

Variable	β (95% CI), *p*-Value
	Univariable Model
BMI	0.098 (0.028 to 0.168); *p* = 0.006
	Metabolic and Inflammatory model
BMI	0.100 (−0.010 to 0.210); *p* = 0.076
Age	−0.017 (−0.154 to 0.120); *p* = 0.808
Male sex	0.156 (0.041 to 0.271); *p* = 0.008
hsCRP	0.327 (0.143 to 0.511); *p* < 0.001
HbA1c	0.194 (0.078 to 0.310); *p* = 0.001
CAD	−0.046 (−0.188 to 0.096); *p* = 0.524
	Cardiovascular Risk Factor Model
BMI	0.033 (−0.089 to 0.155); *p* = 0.596
Age	−0.035 (−0.190 to 0.121); *p* = 0.661
Male sex	0.199 (0.87 to 0.311); *p* < 0.001
HbA1c	0.145 (0.033 to 0.257); *p* = 0.011
CAD	−0.158 (−0.278 to −0.038); *p* = 0.010
LDL cholesterol	−0.089 (−0.216 to 0.038); *p* = 0.170
Hypertension	0.007 (−0.137 to 0.151); *p* = 0.923
Current smoking	0.003 (−0.128 to 0.134); *p* = 0.962

## Data Availability

The data supporting the findings of this study can be obtained from the corresponding author upon reasonable request.

## Abbreviations

BMI	body mass index
CAD	coronary artery disease
CVD	cardiovascular disease
HDL	high-density lipoprotein
HDL_ox_	HDL lipid peroxide content
nHDL_ox_	normalized HDL lipid peroxide content
HFpEF	Heart Failure with preserved Ejection Fraction
HLP	Hyperlipidaemia
IL-6	Interleukin-6
LDL	Low-Density Lipoprotein
LDL_ox_	oxidized Low-Density Lipoprotein
NO	Nitric Oxide
PON-1	Paraoxonase-1
VCAM-1	Vascular Cell Adhesion Molecule 1
